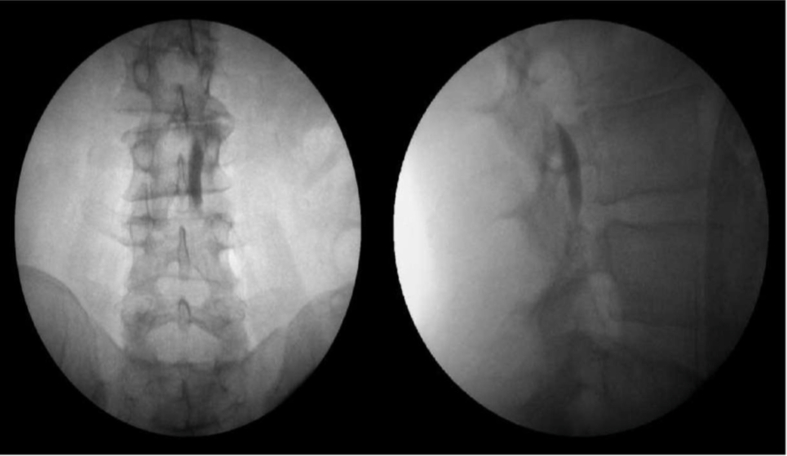# Could this atypical image be an unexpected combination? A visual vignette

**DOI:** 10.1016/j.inpm.2025.100616

**Published:** 2025-07-24

**Authors:** Ridvan Isik, Sena Unver, Savas Sencan, Osman Hakan Gunduz, Serdar Kokar, Kemal Nas

**Affiliations:** a Sakarya Training and Research Hospital, Department of Physical Medicine and Rehabilitation, Division of Pain Medicine, Sakarya, Turkey; b Sakarya University, Faculty of Medicine, Department of Physical Medicine and Rehabilitation, Division of Pain Medicine, Sakarya, Turkey; c Marmara University, Faculty of Medicine, Department of Physical Medicine and Rehabilitation, Division of Pain Medicine, Istanbul, Turkey

**Keywords:** Intradural contrast spread, Dural pulsation, Atypical image

## Abstract

**Background:**

We report the case of a patient who underwent transforaminal epidural steroid injection (TFESI), and a combination of intradural contrast media spread and dural pulsation during the procedure.

**Objective:**

We aimed to raise awareness of the importance of recognising atypical images in interventional pain procedures.

**Methods:**

A 67-year-old woman presented with low back and right leg pain due to spinal stenosis. We performed right L3 TFESI with a Quincke spinal needle under the guidance of C-arm fluoroscopy. The needle placement on imaging consistent with the epidural region, but contrast distribution suggested subdural spread. When we administered contrast material again, the contrast extended and widened a little more in the cranio-caudal direction in the same region but did not disperse. Therefore, we obtained a live fluoroscopic image. The contrast media was accumulated in the same region and showed pulsatile properties in the images. We speculated that this image may be a combination of intradural spread and dural pulsation or may be due to the impact of an artery in the restricted epidural space.

**Results:**

We terminated the procedure.The patient exhibited no neurological deficits, and lumbar MRI and CT angiography were conducted to exclude other causes The neuroradiologist evaluated the examinations and found no abnormalities. To alleviate the persistent pain of the patient, we prescribed medical treatment.

**Conclusion:**

Atypical contrast media distributions may be seen during procedures. To avoid possible complications, it is vital for physicians to have a thorough knowledge of the contrast media distribution pattern.

## 1 Text version of video audio voiceover

Contrast media was accumulated in the same region and showed pulsatile property in the images.

## Declaration of competing interest

The authors declare that they have no known competing financial interests or personal relationships that could have appeared to influence the work reported in this paper.

## Figures and Tables

**figs1:**